# Parental-Bonding and Alexithymia in Adolescents with Anorexia Nervosa, Their Parents, and Siblings

**DOI:** 10.3390/bs12050123

**Published:** 2022-04-24

**Authors:** Stefania Mannarini, Johann Roland Kleinbub

**Affiliations:** 1 Interdepartmental Center for Family Research (CIRF), University of Padova, Via Venezia, 14-35131 Padova, PD, Italy; stefania.mannarini@unipd.it; 2 Section of Applied Psychology, Department of Philosophy, Sociology, Education, and Applied Psychology, University of Padova, Via Venezia, 14-35131 Padova, PD, Italy

**Keywords:** anorexia nervosa, alexithymia, parental bonding, psychopathological symptoms, siblings

## Abstract

Anorexia nervosa (AN) patients and their family-members share alexithymia, anxiety, depression, and other psychological symptoms, in the context of altered attachment. These domains have been individually studied in the context of eating disorders; few attempts have been made to study their interaction, especially including family members. In this study, alexithymia, parental-bonding, and psychopathology were assessed in 32 Italian families consisting of an adolescent AN patient, a sibling, and their parents. We aimed to (a) describe a sample of Italian families with a child affected by AN, notably including siblings; (b) investigate interactions between assessed constructs in patients and their siblings; and (c) investigate possible intergenerational effects. Results showed high alexithymia and psychopathological symptoms in patients but not in siblings, although the latter reported high obsession–compulsion and paranoid ideation scores. Patients’ and siblings’ alexithymia correlated with psychopathology. Parents reported generally low alexithymia. Perceived parental bonding was found to be suboptimal in most participants, yet no clear relationship was found between specific parenting styles and other measured traits, nor did we find any other relevant intergenerational effect. Anorexia nervosa implies psychological difficulties for all family members. Siblings’ psychopathological traits are especially concerning and currently understudied. Implications for future research and clinical interventions are discussed.

## 1. Introduction

Anorexia nervosa (AN) is a severe eating disorder characterized by a distorted perception of body image, restricted food intake, and an intense fear of gaining weight, leading to severe weight loss. In young women, AN has a prevalence ranging from 0.29% to 4.3% [1] and the highest mortality rate of all mental disorders [2]. Furthermore, the prognosis severity of AN is compounded by a comorbidity with other mental health disorders as well as the medical consequences of malnutrition [3,4]. For these reasons, AN is a complex and expensive disease to treat [5], and it represents a serious concern for patients’ well-being and public healthcare.

### Psychological Mechanisms

In the last decade, the characteristics of the AN disorder have been increasingly documented in quantitative and qualitative studies for reviews see [6,7] encompassing detailed observations from the clinical [8], somatic [9], psychiatric [10]), and thought-patterns/schemas [11,12] perspectives.

A recent development in this field of study is the focus on alexithymia. High levels of alexithymia have been reported in AN adolescents e.g., [13] together with greater emotion regulation difficulties and reduced emotional awareness [6]. Beyond recording its mere presence, the trait has been further investigated as a potential mediator of emotional regulation failures. For instance, a previous study [14] investigated 406 female undergraduates reporting that while childhood abuse and neglect are strong predictors of eating disorders, the association between these two variables was fully mediated by alexithymia and depression. These two factors are themselves tightly intertwined, with some authors suggesting that alexithymia is a state-dependent phenomenon in depressed people [15]. Review efforts in the field of eating disorders [16] highlight that alexithymia and depression are often co-morbid psychological health issue, yet literature remains inconclusive on their relationship. Indeed, some of the selected studies report high correlations between the two problems and that group differences in alexithymia scores between patients and healthy controls disappears after controlling for depression. Other studies [17] instead report opposite results such as low correlations between the constructs and that individuals with eating disorders continue to exhibit elevated alexithymia levels even when controlling for general distress.

The role of family is crucial in eating disorder, with growing evidence linking specific familiar traits and dynamics both as determining factors and as consequences of the illness [18].

Specifically, anorexia nervosa has been found to affect family dynamics and relations, including conflicts, disruptions interaction dynamics, limited and divided family life [19]. For instance, a previous study [20] compared the triadic interactions within 20 families of adolescents with AN and 20 families of patients with internalizing disorders. The results showed distinct difficulties in the AN group, specifically in respecting roles, maintaining joint attention and in sharing positive affects.

While being one of the principal domains disrupted by the disorder, families play a pivotal role in managing the disease [21] and intervening on its dynamic can open up important resources aiding patients’ change. For instance, another study [22] showed that the quality of the relationship between 72 adolescents with AN and their parents was clearly associated to the outcome of a 6-month treatment.

Furthermore, since family interactions have a crucial role in supporting the adolescent’s development, various studies started to investigate the link between the emotional functioning of patients and that of their families. One of the main domains of these attempts focused on alexithymia. Studies reported that mothers of anorexic patients resulted more alexithymic than controls [23], although to varying degrees, according to the measure employed. For instance, one study [24] reported that a clinical interview (Toronto Structured Interview for Alexithymia) was able to detect significant levels of alexithymia in parents of AN patients who had previously reported sub-clinical scores using a self-report measure (Toronto Alexithymia Scale, TAS-20). Notwithstanding these difficulties, a study by Gatta and colleagues [25] on 49 psychiatric adolescents and 94 of their parents, found that parents’ TAS-20 scores predicted that of the adolescents, suggesting an intergenerational transmission of alexithymia, both from fathers and from mothers. Moreover, the adolescents’ emotional awareness was affected by the perception of their parents’ parenting styles assessed via the Parental Bonding Instrument PBI [26].

Reinforcing the hypothesis that alexithymia might develop as a developmental response to specific parenting style, another study [27] on families of adolescents with psychiatric symptoms found associations between patients’ alexithymia and their bonding style with parents. Furthermore, these associations were specific for different diagnostic clusters (internalizing vs. externalizing symptoms).

While these results still haven’t been replicated on AN patients, these results could be of particular interest in the field of eating disorders given the interplay existing between childhood experiences of parental bonding or of adversity and vulnerability to an eating disorder [28]. Indeed, higher levels of attachment insecurity across diagnoses are related to greater eating disorder symptoms, and this association is likely mediated by affect dysregulation [28]. For instance, a study by Balottin and colleagues [29] showed that families of AN patients reported peculiar parental bonding attachment patterns, and that the parents’ alexithymic traits, combined with children’s perception of a neglectful parenting style were found to be linked with a higher risk of psychological disorders in offspring [25].

While the functional importance of family in both the severity and treatment of AN is now clearly stated in literature, most of the studies focused on a restricted set of interpersonal dynamics, either mother and daughter dyads or mother, father, and patient triads.

Only recently, scholars started expanding the investigation to other members of patients’ family, and most notably, the role of siblings. Fjermestad et al. [19] interviewed 13 adolescent brothers and sisters of AN patients, reporting how the diseases disrupts these young person’s lives across a broad range of domains, especially relationships with other family members. Siblings of AN patients reported less attention from their parents, and a limited and a divided family life, due to the energy and time required to cope with the illness. Furthermore, these siblings reported increased preoccupation toward food and body image, although not at a clinical level see also [30]. In a similar study on 12 siblings [31], the authors reported how the eating disorder and treatment process put a strain on the sibling relationship. A third study [32] reported how healthy sisters (*n* = 21) of women with ED may also evince more difficulties and avoidance in the recognition of their own emotions as compared to non-related controls, through both self-report and experimental task based on picture of faces. Further recent studies proposed additional qualitative reports of distress in this population [33,34].

These preliminary studies conclude that while not at a direct risk of developing an eating disorder themselves, siblings of AN patients are subject to psychological difficulties. Further investigation is called for to confirm the results and assess whether psychological assistance is needed and in what form for this population.

The scenario drawn by the recent literature presented shows how anorexia nervosa is a complex disorder, escaping the boundaries of the intrapersonal mind of the patient and involving instead the whole network of family relationships, sharing with them dysfunctional traits such as alexithymia and reports of altered attachment relationships. Yet, to the best of our knowledge, no study to date has directly assessed how these domains interact, especially not including an observation of siblings.

The present study has the aims of describing a sample of Italian families with a child affected by AN—notably including siblings—in terms of alexithymia and parental bonding representations. Within this sample, we will investigate the intrapersonal dynamics between psychopathological symptoms, parental bonding representations, and alexithymia in patients and their siblings. Specifically, we hypothesize to observe elevated levels of alexithymia and maladaptive parental bonding styles across all family members, including clinical levels of psychopathological symptoms in patients (especially in terms of depression) and siblings. Furthermore, we expect to find low correlations between depression levels and alexithymia in patients.

Finally, in order to explore the presence of intergenerational dynamics we will study the effect of parents’ alexithymia and children’s perceived parenting style on children’s own alexithymia and psychopathological symptoms. Specifically, we hypothesize that specific parental bonding styles would be associated with more severe psychological symptoms and that higher alexithymia in parents would predict higher levels of the construct in children.

## 2. Materials and Methods

### 2.1. Participants and Procedure

A total of 32 families participated in the study. They were recruited from two clinical centers in northern Italy offering an outpatient rehabilitation program. Given the difficulty to reach and enroll entire families in a research program, we opted for convenience sampling by offering participation to all eligible patients treated at the clinical centers at the time, independently of the advancement of treatment.

Inclusion criteria for families were the presence of an adolescent (age range: 10–24 years, [35]) daughter with a diagnosis of anorexia nervosa and the availability of both parents and a healthy sibling. Exclusion criteria instead were the presence of another major diagnosis (autism, schizophrenia, mental retardation) in patients and lack of Italian language proficiency of any family member. Diagnoses were based on the Diagnostic and Statistical Manual of Mental Disorders, fifth edition, and they were performed in hospital by the psychiatry National Health Service prior to admission to the clinical centers and retrieved from medical records. Information about anorexia subtype was not available.

Families were invited to participate by the clinics’ personnel, informing all members of the research goals and procedures and of the data handling. Written consent for participation was acquired for all participants of age. Finally, participants were administered all questionnaires in separate rooms or at different timepoints.

The research was approved by the Ethical Committee for Psychological Research of the University of Padova, protocol number 2591.

### 2.2. Measures

All family members were administered the Toronto Alexithymia Scale (TAS-20) and the Parental Bonding Instrument (PBI).

The TAS-20 [36] is the most widely employed self-report questionnaire for the assessment of alexithymia. The construct is assessed on three main dimensions: difficulties identifying feelings, difficulties describing feelings, and externally oriented thinking measured through a total of 20 items rated on a 5-point Likert scale. A total score can be calculated from the three scales and interpreted as non-alexithymia (score < 52), possible alexithymia (score from 52 to 60) and proper alexithymia (score > 60). We employed the Italian version of the questionnaire [37].

The PBI [26] is a self-report questionnaire assessing the perceived style of one’s own parents during infancy and adolescence. It consists in 25 identical items for the respondent’s mother and father. Each parent’s style is separately assessed on two dimensions: care and overprotection, representing the dynamics of emotional involvement and of parental control, respectively. By crossing these two dimensions, four possible parenting styles are defined [38] for each parent: optimal parenting (high care—low overprotection) neglectful parenting (low care—low overprotection), affectionate constraint (high care—high overprotection), and finally affectionless control” (low care—high overprotection). The instrument, of which we used an Italian translation [39] showed good stability and reliability.

Furthermore, patients and their siblings were requested to fill the SCL-90-R [40], one of the most widely used clinical self-report scale for research in adults and adolescents (from the age of 13). The SCL-90-R yields a global psychopathology score, called the Global Severity Index (GSI), and 9 subscales: somatization, obsession–compulsion, interpersonal sensitivity, depression, anxiety, hostility, phobic anxiety, paranoid ideation, and psychoticism. The Italian adaptation used showed adequate psychometric properties [41].

### 2.3. Statistical Analyses

Pairwise t-tests with Bonferroni correction were used to highlight differences in the TAS-20 total scores of the different family roles; Pearson correlations were used to explore the relationship between TAS-20 and SCL-90-R scores; ANOVA and Tukey’s HSD post hoc tests were used to compare the average TAS-20 and SCL-90-R scores across the different perceived parenting styles; finally, multiple linear regression modeling was used to study the relationship between parents’ TAS-20 scores and children’s TAS-20 and SCL-90-R scores. All analyses were performed with the statistical software R [42] version 3.6.3. Data will be made available on request.

## 3. Results

The socioeconomic status (SES) was assessed through Hollingshead’s [43] four factor index of social status. On average the participant families had a SES of 37.1 (SD = 9.95; min = 17; max = 56.5). In detail, the SES was low in 2 families (score < 20), medium-low in 8 families (score from 20 to 29), medium in 10 families (score from 30 to 39), medium-high in 11 families (score from 40 to 54), and 1 family had a high SES (score of 56.5).

The parents of 27 families were married, in 3 families they were divorced, and in 1 case the father was a widower. Two families were missing one member at the time of data collection, specifically, one father refused to complete the forms after family enrollment and one mother died. Data of the remaining participants in these two families were retained in the final dataset. Table 1 reports the age, birth order, and sex of all participants. Patients presented an average Body Mass Index (BMI; kg/m^2^) of 16.71 (SD = 2.04; Min = 16.3, Max = 23.2).

**Figure 1:**
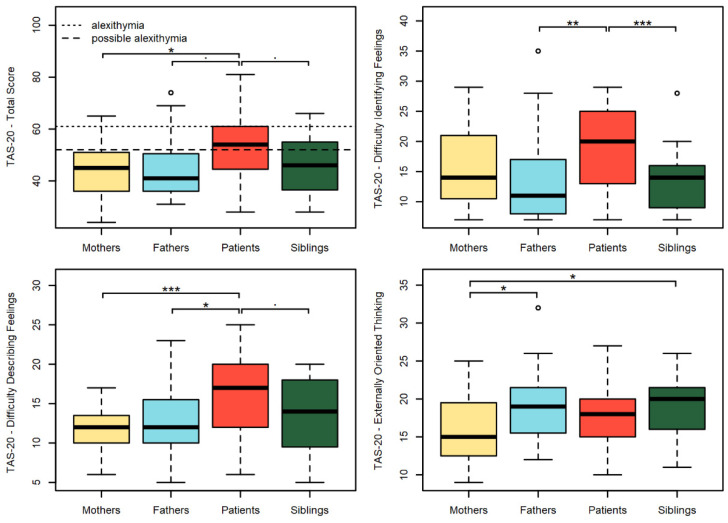
Alexithymia in family members. The boxplots represent the distributions of the TAS-20 scores of the different family members. The significant results of Bonferroni-corrected paired t-tests are reported for the following alpha levels: · = 0.1, * = 0.05, ** = 0.01, *** = 0.001, ◦ = outliers.

Internal consistency for the TAS-20 global score in our sample was satisfactory (Cronbach’s alpha = 0.83). Patients reported the highest levels of alexithymia (Figure 1). Overall, Bonferroni-corrected pairwise *t*-tests showed significant differences in the TAS-20 total score of patients and mothers (*p* = 0.013). In regard to the Difficulty Identifying Feeling subscale (Cronbach’s alpha = 0.87), patients reported scores significantly higher than siblings (*p* < 0.001) and fathers (*p* = 0.003) but not higher than mothers. In the Difficulty Describing Feelings subscale (Cronbach’s alpha = 0.75), patients’ scores were significantly higher than both mothers’ (*p* < 0.001) and fathers’ (*p* = 0.043), while in the Externally-Oriented Thinking scale (Cronbach’s alpha = 0.53; low consistency for this scale is common in literature: [44]) mothers’ scores were found significantly lower than fathers (*p* = 0.025) and siblings (*p* = 0.017). Significant differences at alpha = 0.1, only reported graphically (see Figure 1), concur to describe increased difficulties in patients compared to their families.

According to the instrument’s cut-offs, 25% of patients, 6.25% of mothers, 18.75% of fathers, 16.7% of female siblings, and 0% of male siblings had a score over the clinical cut-off for proper alexithymia, whereas a possible alexithymia score was reported by 31.25% of patients, 15.62% of mothers, 6.25% of fathers, 16.7% of female siblings, and 21.43% of male siblings. Furthermore, a significant difference in average TAS-20 total scores was found between male (μ = 40.93, SD = 10.89) and female (μ = 49.17, SD = 9.48) siblings, t (25.9) = 2.24, *p* value = 0.034.

**Figure 2:**
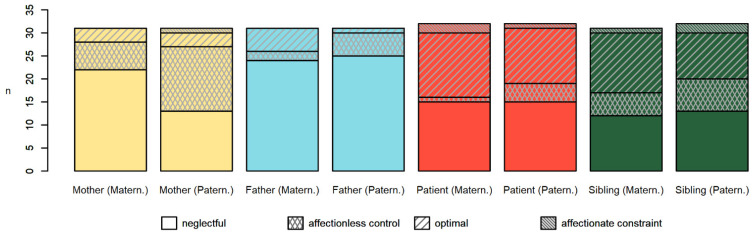
Parental Bonding Inventory in family members. The stacked bar plot represents the proportions of perceived parenting styles coded from the PBI scores. For each participants’ group, the categories distributions are presented both for maternal and paternal relationship (in parentheses).

Internal consistency for the PBI in our sample was satisfactory (Cronbach’s alphas: maternal care = 0.93, maternal overprotection = 0.82, paternal care = 0.91, paternal overprotection = 0.87). The proportions of maternal and paternal parenting style for each participants group are presented in Figure 2. In general, the majority of parents in the study sample reported a perception of having received suboptimal parental care, especially in the neglectful (55.8% of mothers, and 77.8% of fathers, averaging the two respective scales) and affectionless control (31.6% of mothers and 11.0% of fathers) categories. Most patients and siblings instead described their parents’ style as neglectful (47.6% of patients, 39.7% of siblings) or as optimal (41.9% of patients, 37.1% of siblings). Affectionate constraint was the least represented PBI category for all participants, accounting for less than 5% of the sample.

**Figure 3:**
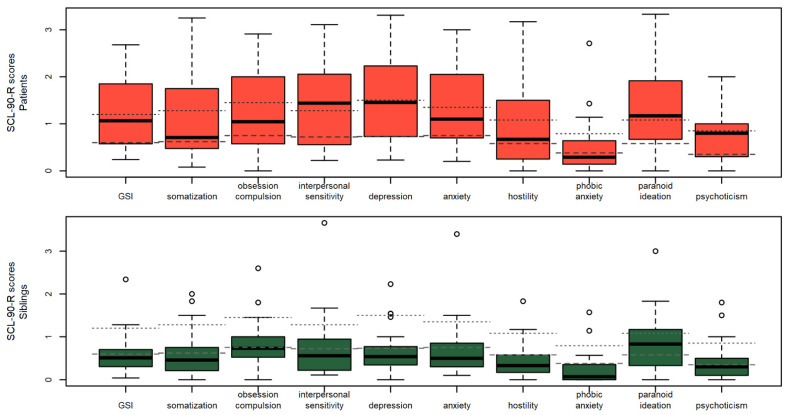
SCL-90-R scores in patients and siblings. The dashed and dotted segments define the cut-offs for moderate and severe symptoms, respectively [45], ◦ = outliers.

Patients’ and siblings’ SCL-90-R scores for the GSI and the 9 subscales are presented in Figure 3 with their respective clinical cut-offs. As expected, patients reported a broad spectrum of psychological symptoms, especially in the interpersonal sensitivity, depression, and paranoid ideation subscales. A total of 43.75% of patients and 9.38% of siblings reported a GSI score above the severely symptomatic cutoff (score > 1.6; [45]).

Only a minority of siblings reported psychological distress in the different scales, with the most severe scores in the domain of paranoid ideation (59.38% were above the moderate symptoms cut-offs). While female siblings had on average higher SCL-90-R scores compared to male siblings, these differences did not reach statistical significance for the GSI or any subscale in uncorrected *t*-tests.

### Trait Interactions in Patients and Siblings

Table 2 shows that alexithymia total scores were correlated with higher symptom scores in most of the SCL-90-R psychopathological domains for both patients and siblings.

Although parents did not report high levels of alexithymia, we tested the hypotheses that variance in that trait predicted patients’ and siblings’ TAS-20 total alexithymia and SCL-90-R scores by means of multiple linear regressions. For both dependent variables, a full model considering all parents’ TAS-20 subscales as predictors, a restricted model considering only mothers’ and fathers’ TAS-20 total alexithymia scores and a null intercept-only model were fit and compared by means of BIC and likelihood ratio tests. Both model comparison strategies favored the null model.

Next, we investigated whether patients’ perceived parenting styles were associated to different TAS-20 total alexithymia and SCL-90-R scores. Given the sparse frequency table of the PBI data for each dependent variable we performed two Bonferroni-corrected t-tests (for the maternal and paternal PBI styles) between the two most frequent categories in patients: neglectful and optimal. The same procedure was repeated for siblings. The analysis revealed that patients who perceived their mothers as neglectful (μ = 59.67, SD = 9.07) had a significantly higher TAS-20 score than patients perceiving their mothers as optimal (μ = 47.42, SD = 13.13), t (23) = 2.9, *p*-value = 0.032. All other comparisons did not allow rejection of the null hypothesis.

## 4. Discussion

The aim of this study was to describe the relationship between alexithymia, parental bonding, and psychopathological symptoms in a sample of 32 Italian adolescents affected by anorexia nervosa (AN) and their families. Specifically, previous literature described alexithymia as a potential mediator of those emotional regulation failures that may underlie AN, independently from other forms of distress such as anxiety and depression [46]. Other studies focusing on a broader spectrum of psychiatric disorders found that such mediating effect of alexithymia in patients may in turn be regulated by parents’ alexithymia as well as specific styles of parental care received by parents [25].

Additionally, this study aimed to further expand the understanding of AN family dynamics by offering a first quantitative report on whether these dysfunctional traits are shared by the siblings of AN patients as well. 

### 4.1. Findings of the Current Study

The results showed that more than half of patients reported TAS-20 scores above the possible alexithymia cutoff. Patients’ scores were significantly higher compared to the other family members, especially in the difficulty in describing and identifying feelings scales. These results are in line with previous literature [47], highlighting how alexithymia is indeed a crucial dimension of the psychological mechanisms underlying AN without exception in our Italian sample. Regarding psychopathological symptoms, the large majority of patients reported moderate to severe scores in the GSI (75% above cutoffs) and most other SCL-90-R scales, especially in the interpersonal sensitivity (75% above cutoffs, µ = 1.41, SD = 0.88), depression (84.37% above cutoffs, µ = 1.56, SD = 0.91), and paranoid ideation (87.5% above cutoffs, µ = 1.36, SD = 0.83) dimensions. As described in the introduction, various authors e.g., [46,48,49] propose a functional independence between alexithymia and other psychopathological symptoms and especially anxiety and depression. In our sample, though, apart from phobic anxiety and hostility, all SCL-90-R scales were found associated to alexithymia scores with moderate correlations ranging from r = 0.238 to r = 0.448. These associations add evidence toward previous literature e.g., [32] reporting depression as the main explanatory factor for TAS-20 score differences. Our study though did not assess difference in emotion regulation strategies in patients, so more sophisticated conceptualizations regarding this debate [46] could not be tested.

In regard to parental bonding, patients’ reports were mostly split between optimal and neglectful parenting styles, with the latter (referring to maternal bonding) predicting significantly higher TAS-20 scores than the former. The only patient reporting an affectionless control maternal caring style had a significantly higher SCL-90-R GSI score.

As for parents, mothers and fathers reported relatively lower alexithymia scores, with only approximately a quarter of each group (22.1% and 25.6%, respectively) reporting TAS-20 scores above the clinical cut-offs. Mothers reported particularly low scores in externally oriented thinking in comparison to fathers and siblings. 

It is important to note, though, that the low levels of alexithymia in parents could at least partially be an effect of the self-report nature of the TAS-20 instrument. Indeed, in an aforementioned study [24], we found that parents of daughters with anorexia nervosa reported low levels of alexithymia with the TAS-20 instrument, whereas much higher levels could be detected through the Toronto Structured Interview for Alexithymia. This could be caused either by misperception of parents’ own difficulties or by biased self-report answers possibly elicited by the social pressure and sense of guilt and responsibility associated to parenting a child affected by AN [50].

Adolescent brothers and sisters of our study’s patients reported TAS-20 scores in line with that of their parents. Only about a quarter of the sample (28.13%) reported scores above the cutoff for possible alexithymia, and the sample mean was µ = 45.56 (SD = 10.79). The result is very similar to that of another study reporting alexithymia levels in siblings of eating disorders patients [32] that reported comparable average TAS-20 scores, albeit their sample consisted only of female sisters and the authors did not differentiate between sisters of AN and bulimia nervosa patients. Regarding gender, we found that female siblings reported significantly higher TAS-20 total scores. While we lacked the power to analyze the gender effect on this data, the significant differences observed in the TAS-20 subscales, in comparison to patients, seemed to be mostly driven by male siblings. This may suggest that female siblings of patients affected by anorexia nervosa are particularly vulnerable regarding emotional regulation. We suggest that further studies on AN siblings may specifically focus on a gender effect.

While not as severe as in patients, most siblings reported a range of psychopathological symptoms on the SCL-90-R questionnaire. The obsession compulsion (µ = 0.81, SD = 0.49) and paranoid ideation (µ = 0.82, SD = 0.63) were the two subscales where most siblings reported scores above the clinical cutoffs (43.75% and 59.38%, respectively), and no significant difference was found between male and female siblings. Results of the PBI questionnaire were similar to that of patients, with optimal and neglectful as the most recurring parental styles. Furthermore, similarly to patients, siblings who perceived their mothers as affectionless controlling had a higher SCL-90-R GSI score, a result which enforces the literature reporting controlling maternal behaviors exacerbating family conflict and symptomatology.

On the whole, these findings confirm previous preliminary reports e.g., [19] highlighting how AN implies a burden on the lives of patients’ siblings. While the relatively low percentage of clinical alexithymia in siblings might hint toward a degree of resilience in these adolescents, the specific psychopathological symptoms, and the correlations between alexithymia and the SCL-90-R subscales highlight a pattern of potential criticality. High scores on the paranoid ideation scale were particularly worrying, as the scale measures thought alterations and symptoms of projective thinking, hostility, suspiciousness, and a fear of loss of autonomy. An alternative interpretation of this scale high scores is offered by Cimino and colleagues [51] who suggest that some of the paranoid ideation scale items, in the context of eating disorders, may not reflect thought alterations but actual difficulties. For instance, items such as “Feeling others are to blame for most of your troubles” or “Feeling that you are watched or talked about by others” might be concrete experiences of an adolescent sibling of a psychiatric patient, more than altered reality testing.

Notwithstanding this hypothesis, on the whole the results lead to the conclusion that siblings of AN patients may be at risk of moderate to severe psychological distress, and corroborate previous suggestions to include siblings in family-based treatments or even to develop specific interventions [19,52].

Previous studies on intergenerational dynamics in psychiatric patients [22,25] drew a picture where parents’ alexithymia, parental bonding styles, and interactions between parents’ alexithymic traits and patients’ perceived parental styles could influence the severity of psychological distress. Given that AN patients were described to report peculiar PBI styles [29], we hypothesized that these intergenerational effects could be present in this sample, too. Our results confirmed that AN families present specific PBI styles patterns, and that especially a controlling style in mothers was associated to heightened distress in children, yet we found no support for any intergenerational effect on the measured dimensions in our sample.

### 4.2. Strengths and Limitations

The present study provides one of the first in-depth examinations of various psychological dimensions: alexithymia, parental bonding styles, and psychopathological symptoms in AN families, with the notable inclusion of adolescent siblings of both sexes. This innovative approach allowed for directly addressing theoretical questions that were previously only discussed speculatively. Previous findings were mostly confirmed in this study’s families, reporting high levels of alexithymia and psychological distress in patients and less so in other family members.

Finally, some limitations can be found in our study. First of all, while the overall number of participants (*n* = 126) isn’t small, the within-group numerosity (*n* = 32) might have been too small to detect specific effects, especially in the more complex intergenerational analyses. For instance, some of the PBI categories were underrepresented or completely missing in some groups. Similarly, further studies on larger samples could explore how siblings’ involvement, both as a resource and as a vulnerable family member, is moderated by their gender. Furthermore, our diagnostic data did not differentiate between purging and restricting types of AN, which may involve alexithymic traits in different ways [16]. Lastly, the self-report nature of the TAS-20 instrument may have led to an underestimation of alexithymia, especially in parents [24], whereas clinical interviews or experimental tasks may prove more sensitive in assessing this dimension.

### 4.3. Clinical Implications

The present quantitative results confirm previous qualitative research expressing concern about the psychological wellbeing of siblings, although more studies are warranted to disambiguate possible confounders. Finally, while the results reiterate how AN involves the whole family in a complex dynamic, the hypotheses of intergenerational associations between the measured constructs were disconfirmed for our sample.

### 4.4. Future Directions

Further studies are warranted to replicate and extend these findings with other psychological dimensions and assessment methods. Specifically, upcoming research should consider adopting alternatives to self-report assessment tools, especially for alexithymia. Concerning the intergenerational hypotheses, further studies should aim to collect larger samples, allowing for more sophisticated analytical approaches such as network analyses.

## Figures and Tables

**Table 1:** Demographic data.

Family Role	Age (Years)				Birth Order				Sex	
	Mean	SD	Min	Max	I	II	III	IV	F	M
Mothers	50.1	4.52	41	58					31	-
Fathers	52.9	4.73	41	64					-	31
Patients	18.2	2.60	14	24	14	15	2	1	32	-
Siblings	18.4	4.00	12	25	15	13	4	0	18	14

Note: The table reports the age and the sex of all participants, divided by their family role. The birth order of children is reported as well.

**Table 2:** Pearson correlations between TAS-20 total scores and SCL-90-R scales.

	Patients		Siblings	
	*r*	*p*-Value	*r*	*p*-Value
GSI	0.574	<0.001 ***	0.399	0.024 *
Somatization	0.511	0.003 **	0.352	0.048 *
Obsession–compulsion	0.593	<0.001 ***	0.238	0.189
Interpersonal sensitivity	0.575	<0.001 ***	0.354	0.047 *
Depression	0.504	0.003 **	0.339	0.057
Anxiety	0.487	0.005 **	0.448	0.01 *
Hostility	0.282	0.118	0.331	0.064
Phobic anxiety	0.087	0.637	0.323	0.071
Paranoid ideation	0.624	<0.001 ***	0.420	0.017 *
Psychoticism	0.494	0.004 **	0.356	0.045 *

Note: * *p* < 0.05; ** *p* < 0.01; *** *p* < 0.001.

## Data Availability

The data presented in this study are available on request from the corresponding author.
